# Clinical dyspnea scenario: Using high-fidelity situation simulation teaching program to evaluate learning effectiveness for clinical junior and pre-clinical nurses

**DOI:** 10.3389/fpsyg.2022.1015106

**Published:** 2023-01-09

**Authors:** Yu-Hsin Liu, Yi-Maun Subeq, Po-Han Lin

**Affiliations:** ^1^Department of Nursing, China Medical University Hospital, Taichung City, Taiwan; ^2^Department of Nursing, National Taichung University of Science and Technology, Taichung City, Taiwan; ^3^Cardiovascular Surgery Division, Taichung Veterans General Hospital, Taichung City, Taiwan

**Keywords:** dyspnea, high-fidelity situation simulation, learning effectiveness, nurse post graduate year, pre-clinical nurses

## Abstract

**Background:**

Confronting a patient’s breathing difficulties, clinical junior nurses often do not know how to respond, and fail to give proper evaluation and treatment. Sudden changes in the condition make the clinical nursing novices feel pressured, and even, frustrated.

**Objectives:**

This study aims at exploring the effectiveness of the high-realistic situational simulation of dyspnea teaching program for pre-clinical and clinical 1^st^ year nurses after graduation.

**Design:**

This study adopts a quasi-experimental repeated measure pre-post-test design study with nonequivalent control group pre- and post-test research design. A total of 135 subjects participated in the research: nurses, post graduate year (NPGY) (*N* = 69), have been employed in the adult ward of a medical center for less than 1 year; and pre-clinical nurses (*N* = 66), 3rd-year nursing students with nurse licenses from a university in the central part of Taiwan. Simulation-based education instructed and incorporated into the high-realistic situation simulation dyspnea teaching program. Questionnaires were used to measure the effectiveness of learning, data were analyzed with SPSS version 20.0, and the scores were repeatedly measured with the generalized estimating equation.

**Results:**

For “cognition, skills, attitude, self-efficacy, teamwork,” NPGY and pre-clinical nurses’ post-tests are better than pre-tests, with statistically significant results. NPGY nurses’ “skills,” “attitude” and “teamwork” learning effectiveness are better than those of the pre-clinical nurses.

**Conclusion:**

The high-realistic situational simulation of dyspnea teaching program can significantly improve the learning effectiveness of NPGY nurses and pre-clinical nurses in the clinical evaluation and treatment of dyspnea.

## Introduction

With the rapid development of medicine and technology, the acute severity of inpatients has increased. Nursing staff monitor patients 24 h a day. To interpret monitoring instruments and conduct physical assessments, these techniques should be significant for the nurses. Still, they should hold technical operation skills and drug knowledge, and complete various complex tasks in time. In treatment, interventional measures can be adjusted at any time according to the patient’s safety conditions (Benner et al., 2010). When a case of dyspnea occurs, the first line of contact with the patient is the NPGY nurse. Commonly, clinical practice nurses might not know how to respond when dyspnea occurs, and fail to give appropriate intervention. Such a sudden condition may make the NPGY nurse feel pressured, or the nurse may even fail to recognize changes in the patient’s condition, causing potential patient safety incidents (Huang et al., 2016).

## Related work

### The importance of situational simulation in nursing education

In recent years, situational simulation education has been applied to medical-related education research, and a consensus international situational simulation teaching remains an effective learning strategy (INACSL Standards Committee, 2016). Some research results, in Taiwan and abroad, support realistic teaching not only to improve the knowledge of nurses and nursing students, but also strengthen learning motivation, deepen learning impression, improve students’ skill efficiency, self-confidence, completeness of situational awareness, teamwork, and anxiety reduction (Bland et al., 2014; Khalaila, 2014; Kapucu, 2017). Norman (2012) completed a systematic literature review of nursing simulation education from 2000 to 2010, and found, in 32 articles, situational simulation teaching can assist students in developing communication skills and patient safety and avoiding medication errors. Such teaching cultivates nurses’ knowledge, skill, safety, and self-confidence (Norman, 2012).

### Novice nurses’ clinical competency and training strategy

The clinical situation is ever-changing. When graduates enter the clinic, they feel unable to respond to sudden changes in the disease, feel frustrated and anxiety, and even make improper team communications. Failure to identify changes in the patient’s condition might cause patient safety incidents (Lewis et al., 2012). The in-service education of NPGY nurses adds to the knowledge based on classroom/online teaching, and is supplemented by one-on-one clinical teaching. Novice clinical NPGY nurses still lack practical experience and cannot adapt to the clinic, so as to leave the workplace earlier (Lai et al., 2014). A former study regarding the effects of teaching expenses subsidy pointed out that the self-assessment of novice nurses in the “Linking School Education to Clinical Work” was only 60%, and the “Clinical Practice Ability Improvement” was only 60%. The proper communication ability of team members, patients, or family members of patients reached only 70%. This shows that there remains room for improvement in the effectiveness and clinical practice ability of novices (Chang et al., 2017), therefore changing the teaching mode enables new recruits to improve their practical abilities and communications. Nursing education cultivates high-quality nursing staff members. Regardless of the re-scientization of medicine, human behavior and patients’ situations are ever-changing. The work of the nursing staff is related to patients’ survival, with no minor mistake allowed. The nursing staff earns experience from various case situations, interacting with the case, family, and even the nursing team. Such experiences are converted into knowledge, giving the staff ability to judge, guide them to quickly grasp the situation, and predict what may happen (Wu et al., 2017). Nursing education in Taiwan is diverse and complex. Problems with different academic courses are the gap between theory and practice, inadequate clinical reasoning education for nursing graduates, insufficient practical ability of nursing freshmen, and low adaptability, high turnover rate challenges (Lai et al., 2014). Literature suggested that the mood change (i.e., anxiety) impacted novice staff’s performance. Similarly, Lawrence et al. (2014) posited that beginning learners with anxiety conditions may be inefficient in skill learning. Furthermore, Lawrence’s study indicated that the earlier in learning the mood is associated with the task, the greater the performance will be in relation to that mood being present in future. As such, the researchers concluded that training with anxiety should be a role for eliminating learning shock and practitioners should aim to duplicate such experiences in real high-pressure circumstances, so as to provide an effective training environment (Lawrence et al., 2014).

### High-realistic situation simulation education and learning effectiveness

The International Nursing Association for Clinical Simulation and Learning defines simulation as a teaching strategy that is created or duplicated similar to what might happen in real life (Ross and Carney, 2017). In recent years, simulation teaching has been widely used in the clinical training of western medical doctors and other professional personnel like nurses, dentists, and pharmacists (Camp and Legge, 2018). Simulation can be incorporated into one or more forms to promote, improve, or verify participant performance (INACSL Standards Committee, 2016).

The traditional classroom teaching, teaching, and replying to teaching might be the same instructional model in the classroom for students to learn (Norman, 2012). Thus, students and novice nurses should acquire needed knowledge, skills, and behaviors to deal with challenges or/and difficulties in clinics. Still, students caring for complex health problems of patients frequently fail to clasp efficient tips so that frustration and inefficient learning might be detected. Stress and anxiety are experienced because of lacking of familiarity with complex and advanced technological tools and equipment, increased medical errors, and fear of making mistakes (Ross and Carney, 2017). Studies explained that the use of high-fidelity simulation methods in nursing education may positively affect on the development of students’ knowledge, skills, and attitudes (Eyikara and Baykara, 2018), correct techniques of administering intra-muscular injections to prevent complications (Smallheer et al., 2018), students’ knowledge, skill levels for cardiac auscultation are increased, yet anxiety reduced (Vural Doğru and Zengin Aydın, 2020), and improving critical care knowledge (Filomeno et al., 2020).

Teamwork and communication have been identified as vital components of safe healthcare systems. Prior studies across several industries have recognized simulation as an effective way of improving these skills, particularly in the acute care setting, and require efficient collaboration (Gjeraa et al., 2014). As Lewis et al. (2012) specified in their study, reviewing 16 related articles on immersive situation teaching, they found that immersive situations can effectively improve nurses’ handover communication skills and the medical team behavior in crisis cases. Armenia et al. (2018) synthesized some articles of high-fidelity team-based simulation in acute care settings. They pinpointed the use of high-fidelity simulation with team-based has lingering in acute care, like intensive care unit, and trauma teams (Armenia et al., 2018).

Still, some research findings indicate that high-fidelity situation simulation may positively impact on student self-efficacy, learning satisfaction, psychomotor skills, and critical thinking (Aebersold et al., 2018). Scenario simulations can provide critical care situations that allow nurses the opportunity to employ and integrate their knowledge and skills so as to apply to patient cares (Boling and Hardin-Pierce, 2016). Such scenarios simulate the pressure that allow nurses to experience during critical care. So, team cooperation among nurses and healthcare staff is found, confidence has been developed (Cristallo et al., 2021). Also, a simulation course focusing on interaction strategies to enhance learner self-efficacy should be beneficial (Chang et al., 2022). Camp and Legge (2018) found, in their reviewed articles, that simulation education programs of realistic situations could improve students’ self-efficacy, skill proficiency, clinical reasoning, collaboration and leadership, communication and cooperation, and increased self-confidence.

Students who care for patients with complex health problems, such as dyspnea, often fail to grasp the key points. Therefore, frustration and inefficient learning emerge. The immersive teaching is to design a simulated environment similar to the clinical environment and the patient situation. In this immersive situation, the students try to perform an assessment and treatment or solve a clinical problem. Thus, the advantage of realistic situational teaching provides students with a safe learning environment where they can practice and refine various skills. Since it is operated in a realistic situation rather than directly on the real patient, failure in the process does not endanger the safety of patients. The process can also be practiced repeatedly, depending on the learning status of the students, and there are no time limitations (Huang et al., 2016).

### Dyspnea situation through high-fidelity

Dyspnea increases breathing frequency, causes changes in the depth of the respiratory rhythm, cyanosis, use of respiratory assist muscles, pout-style breathing, and daily activities reactions such as reduced function, nervousness, depression, insomnia, fatigue, and social isolation. It causes breathing difficulties of six types: pulmonary, psychogenic, neuromuscular abnormalities, blood-related, poisoning, and mental illness (Chang et al., 2015; Chen et al., 2016; Fuessl, 2016). In order to realize the integration of teaching, examination, application, and certification, practice-oriented nursing teaching has become the leading trend. As proved, for improving cognition and skills, realistic teaching can facilitate communication and teamwork. In view of this, it is necessary to develop a suitable program that simulates the real dyspnea situation through high-fidelity, and guides students to learn how to perform clinical assessment and treatment of dyspnea.

This study aims at exploring the effectiveness of the subjects’ cognition, skills, attitudes, self-efficacy, teamwork, and the effectiveness of NPGY and licensed pre-clinical nurses. In the past, comprehensive nursing education was rarely used for clinical assessment and treatment training of critical symptoms. Related studies that comparing the difference in using high-fidelity simulation on pre-graduation nursing students and newly-registered nurses were none. Still, the ability of both groups in dealing with patients’ dyspnea was limited owing to their in-clinic experience and knowledge. After intervention, using the “Highly Realistic Situational Simulation of Dyspnea Teaching Program,” the research results would provide relevant references for future instructional design and on-the-job education for new clinical personnel.

## Materials and methods

### Study design and setting

This quasi-experiential study measures, through a pre-post-test design, with a convenient sampling, 135 participants were recruited. Sixty-nine first-year NPGY nurses are serving in adult wards in a medical center, and 66 3rd-year licensed nursing students were recruited from a university. The ethical review has been approved as CMUH108-REC3-067. Initially, we highlighted the purpose and methods of the research, and announced the course time of the workshop in the hospital or school to recruit participants. First, research participants signed the consent form to take part in the research. Before the teaching program started, demographic data were collected: age, gender, education level, and clinical work department. Then, we use “Clinical Assessment and Management Cognition and Attitude Structured Questionnaire,” “Objective Structured Clinical Test for Clinical Assessment and Management of Dyspnea (OSCE),” “General Self-Efficacy Scale,” and “Team Cooperation Observation Scale (TPOP)” and proceed with the pre-test to understand their previous cognition, skills, attitudes, and self-efficacy and inter-professional team cooperation. After the pre-test data were collected, the “high-realistic situational simulation of dyspnea teaching” was carried out. In order to prevent different teachers from interfering with the research results, the NPGY student group and the nursing group were led in the “High-realistic situational simulation of dyspnea teaching workshop” with the same instructor. Two weeks after the course, subjects filled out the questionnaires.

### Curriculum design and development of the dyspnea teaching program

#### Analyze learner

The trainees are NPGY nurses and college senior nursing students. Based on the announcement of the Taiwanese Ministry of Health and Welfare, NPGY has earned the nurse practitioner license and is registered in the working place. The new nurse practitioners who have worked within 2 years need to be trained and improved in various clinical care capabilities. Pre-graduation nursing licensed students are those who still study in a university, not yet working in a clinic. They have completed basic nursing, biomedicine and disease care, and professional courses. These senior students, who graduated from a five-year junior college, are licensed nurse practitioners. At school and hospital practice fields, they have not received high-realistic situation simulation dyspnea assessment and clinical treatment-related courses.

#### Set learning goals

Use the eight nursing core aspects by proclamation of 2010 Taiwan Nursing Education Evaluation Committee: Critical thinking skills; General clinical nursing skills; Basic biomedical sciences; Communication and teamwork; Care; Ethical literacy; Duty; Lifelong learning (Chung, 2000). The core competence is cultivated for this lesson plan. The learning objectives are drawn up based on the eight core competencies, and observations as well as interviewing clinical NPGY nurses’ experience in caring for patients with dyspnea. Further, we also refer to relevant domestic and foreign literature for teaching goals, setting up the teaching goal of a “highly realistic situation simulation dyspnea teaching program.”

#### Drafting teaching guidelines for dyspnea in high-realistic situations

The teaching guidelines are based on the above eight core learning goals of nursing that include: an overview of dyspnea simulating situations, the purpose of teaching plans, basic information on standardized roles, and summary of conditions, props and equipment, and standardized role-guide scripts.

#### Selection of teaching resources

Based on the concept of high-fidelity situational simulation teaching to develop a number of teaching resources, including independent audio-visual teaching and written materials, SimMan 3G, standardized family members, and standardized physicians.

#### Textbook production

(A) The teaching notes for dyspnea, including the concept and definition of dyspnea, respiratory system physiology, oxygenation supply and demand balance (ventilation, diffusion, carrying, perfusion), cause of dyspnea and clinical reasoning, dyspnea diagnostic examination, dyspnea assessment tool, and clinical treatment of dyspnea; (B) Conceptual diagram of clinical reasoning for dyspnea; (C) Flow chart of clinical treatment of dyspnea.

### Simulation planning and implementation

#### Self-study one week before class

Researchers use QR code links and URLs 1 week before providing students with self-learning materials for dyspnea assessment and clinical treatment teaching materials, including: (A) dyspnea teaching handouts; (B) respiratory system body assessment audio-visual teaching; (C) dyspnea clinical reasoning concept map; (D) flow chart of clinical treatment of dyspnea, providing students with independent learning.

#### High-realistic situation simulation of dyspnea teaching

##### Lecture and assessment

**Figure 1:**
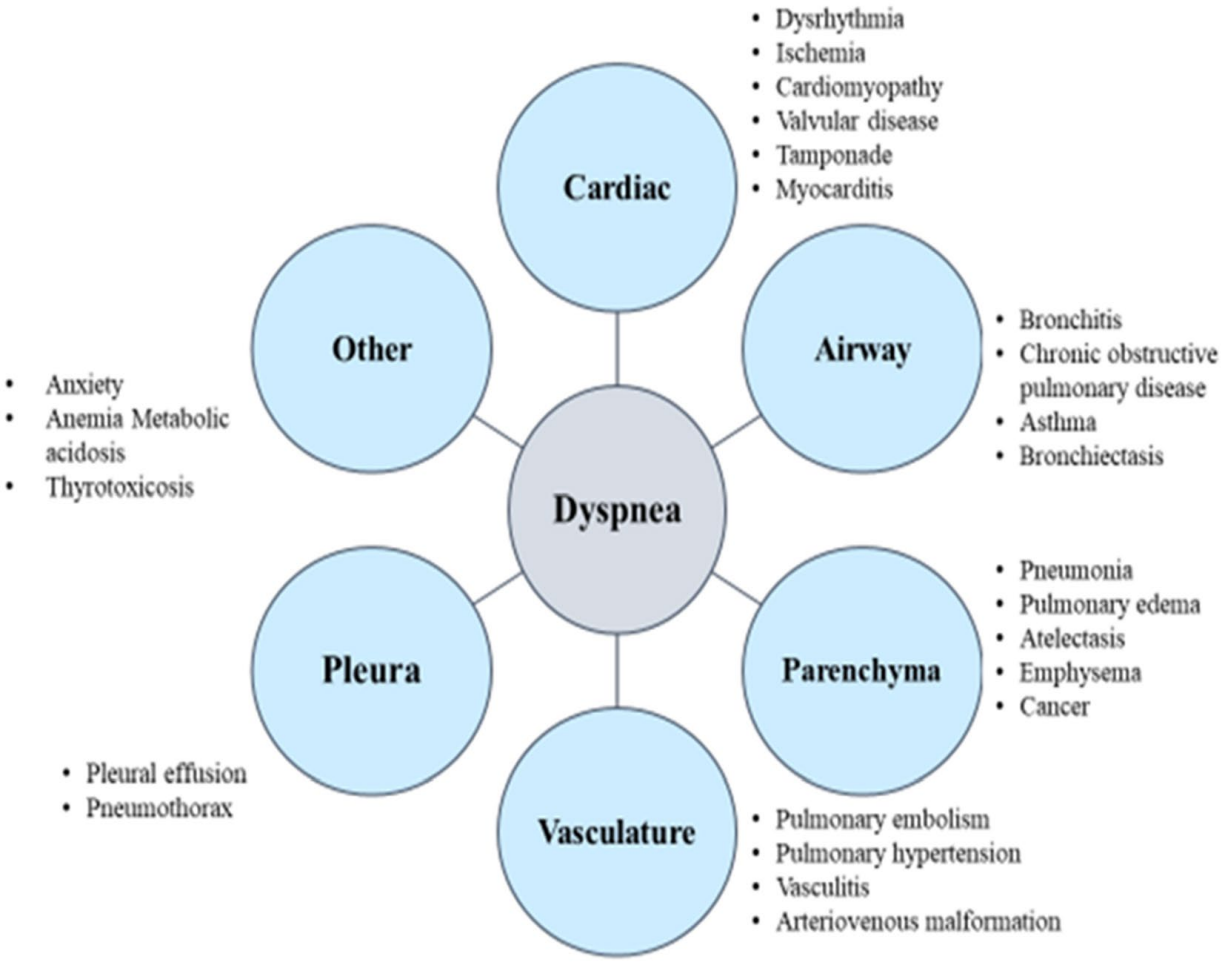
Dyspnea clinical reasoning concept map.

**Figure 2:**
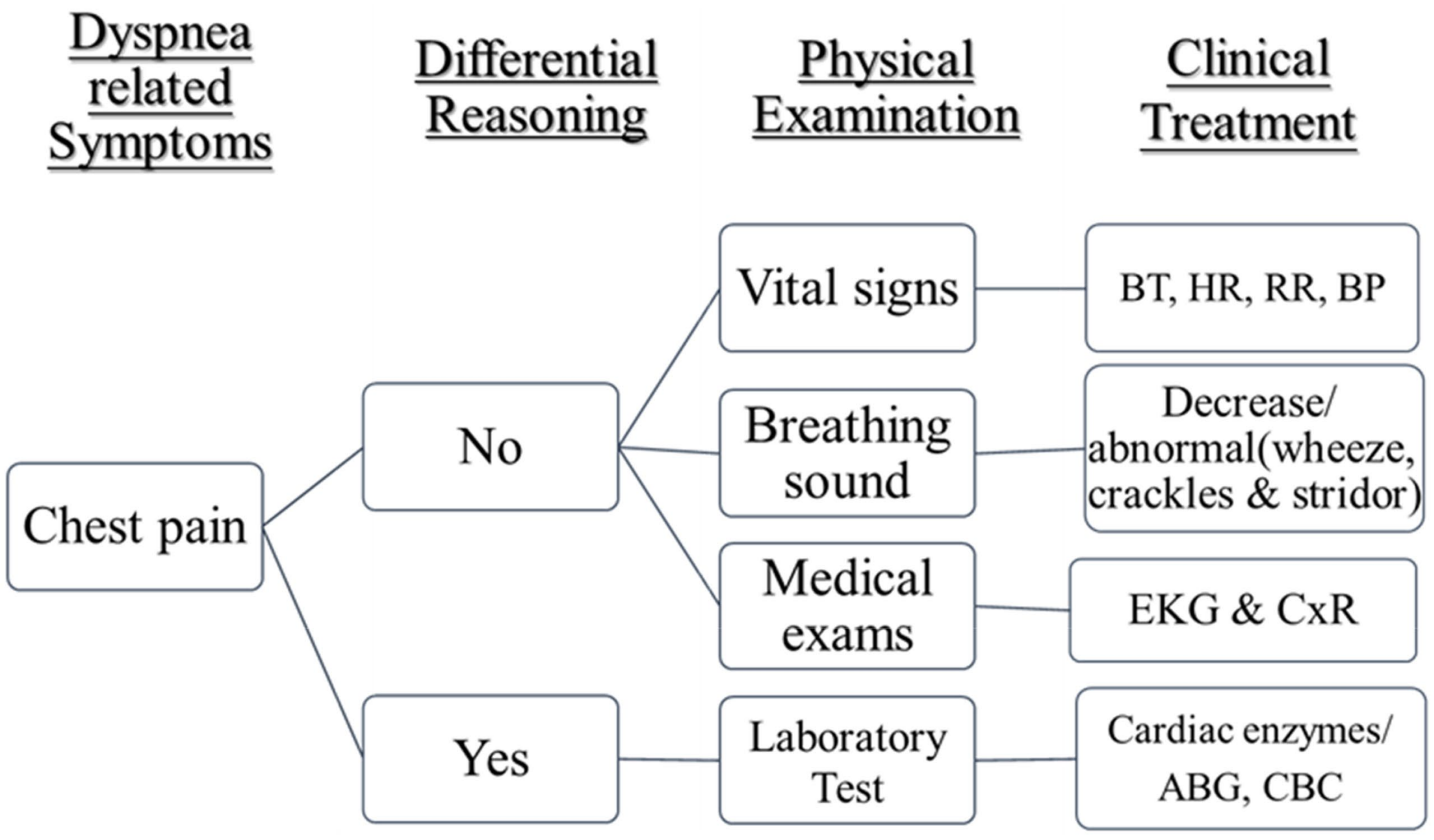
Dyspnea clinical treatment flowchart (Parshall et al., 2012).

The first and second lectures will be conducted, with teaching on dyspnea assessment and clinical treatment concept reasoning: to be carried out in the first and second period before class, need to perform assessment (pre-testing). The researchers will lead the trainees to conduct dyspnea assessment and clinical treatment conceptual reasoning, teaching according to the “Dyspnea Teaching Notes” and “Dyspnea Clinical Reasoning Concept Map” and “Dyspnea Clinical Treatment Flowchart.” In the past, patients’ dyspnea evaluation has been related on the search for corresponding pathophysiology. Some medical causes of dyspnea are provided in Figure 1 (American Thoracic Society, 1999) and clinical treatment of dyspnea in Figure 2. However, Dyspnea Clinical Treatment Flowchat (Figure 2; Parshall et al., 2012) was guided for those who participated to analyze dyspnea-related symptoms and to recognize the assessment of dyspnea for patient evaluation and management.

##### Practice and debriefing

Lessons 3 and 4, conducting “High-realistic simulation of breathing difficulties situation practice and debriefing.” Researchers discuss the process and use big-character posters to provide for team members (6–8 people will be randomly organized) to write down the dyspnea treatment process and practice realistically with SimMan 3G high simulation. Students actually participate in the operation to increase their familiarity with the process of dyspnea assessment. In addition, standardized family members and standardized doctors have simulated standard scenarios for dyspnea (spontaneous pneumothorax, acute myocardium) to conduct a scenario simulation exercise, and observe the operation of the trainees with the “Objective Structured Clinical Test for the Clinical Assessment and Treatment of Dyspnea.”

Based on the simulated scenarios (Table 1), those who participated in the study will make a decision for the clinical physiological status and test data of SimMan 3G from key events so as to make proper interventions and communication with standardized family members at the same time, as well as to cooperate with professional teams effectively. For evaluation of performance in their group, team members should follow and complete tasks, including: (A) distinguish the relationship between abnormal vital signs and Dyspnea, (B) correctly perform the assessment of the patient’s chest cavity and breath sounds, (C) correctly interpret ABG, CBC, Lab Data, and determine the clinical significance of abnormal values related to Dyspnea; (D) based on the Dyspnea concept map, use clinical thinking skills to differentially diagnose the causes of dyspnea; (E) provide appropriate nursing intervention for Dyspnea cases; (F) show empathy, respect, and care for patients with dyspnea and their families; (G) to communicate and collaborate with inter-professional medical teams and to take care of dyspnea cases together; (H) to demonstrate their professional confidence.

**Table 1:** Simulated scenarios.

Scenario 1: Pneumothorax				
Mr. Li Daxiong, 19 years old, BH 190 cm, BW 50 kg, has a history of smoking for 3 years, has a cold and fever in last few days, has been taking cold medicine for 3 days, and cold symptoms has improved. “I felt pain in the left chest”, which improved slightly after resting in the health room. After returning home, the pain in left chest gradually intensified, but it did not improve after rest, and I felt shortness of breath. The mother accompanied Mr. Li to the outpatient clinics. It is recommended to be admitted to the internal medicine ward for observation and treatment.				
**Key events**	**Learning objectives**	**METI MAN (simulators)**	**Standardized family/medical staff**	**Medical props or equipment**
Symptoms: shortness of breath, dyspnea and left chest pain	1. Taking history	1. Condition setting: BT:36.0, HR 124/min, RR35/min, BP165/62 mmHg	Anxiety, Unconcerned, Caring, etc.	O_2_ therapy, Physical monitors
	2. Data collection for Dyspnea	2. Medical exam: EKG 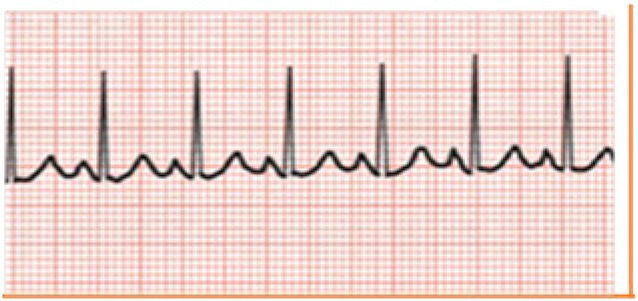		

After the scenario simulation practice, the researchers conduct group discussion and debriefing. If questions are proposed, they will immediately resolve their questions or/and doubts so as to improve the learning effectiveness of the students.

#### Learning effectiveness evaluation—Pre-post-test survey

The pre-post-test was conducted in the clinical skills classroom of a medical center and a nursing university in the central part of Taiwan. Demographic information included age, gender, and education level; and the clinical nursing group adds investigation clinical work departments and nursing clinical qualifications. Two weeks after the completion of the high-realistic situation simulation dyspnea teaching course, post-tests were performed in the clinical skills OSCE classrooms of a medical center and a nursing university. The contents of both the pre- and post-test remain the same.

### Cognition and attitude

Clinical assessment and treatment of the dyspnea “cognition” measurement table: referenced multiple documents and compiled according to the content of the teaching plan, a total of 25 questions. The higher the score, the better the awareness of clinical evaluation and treatment of dyspnea (Chang et al., 2015; Chen et al., 2016; Fuessl, 2016). “Attitude” scale for clinical management of dyspnea is compiled with reference to much domestic and foreign literature, mainly assessing the attitudes of the nursing taff to the clinical assessment and treatment of dyspnea, with a total of 15 questions. The total score is between 15 and 60 points; the higher the score, the more positive the attitude toward treatment. The more positive attitude indicates the more capable of clinical handling of dyspnea.

### The formative clinical skill

Compiled with reference to domestic and foreign literature to design Objective Structured Clinical Test (OSCE) Scale for Clinical Evaluation and Management of Dyspnea, nursing staff’s ability to perform clinical assessment and treatment of dyspnea were evaluated, 18 items in total. The scores are, in order: “completely achieved” (3 points), “mostly achieved” (2 points), “partially achieved” (1 point), “not achieved” (0 points). In order to pass, 38 points or more should be reached. The higher the score, the more correct the treatment of dyspnea.

### General self-efficacy

This research uses the “General Self-Efficacy Scale (GSES)” developed by Matthias Jerusalem and Ralf Schwarzer in 1981 as a tool (Schwarzer and Fuchs, 1995), and the Chinese version of the “General Self-Efficacy Scale” developed by Zhang and Schwarzerc (1995). A total of 10 questions is used with a Likert 4-point scale scoring: 4 points for “completely correct,” 3 points for “mostly correct,” 2 points for “fairly correct,” 1 point for “completely incorrect” (Schwarzer and Fuchs, 1995).

### Teamwork skills

TeamSTEPPS Performance Observation Tool (TPOP), is a set of evaluation systems created by the US Agency for Healthcare Research and Quality (AHRQ) and the US Department of Defense, and it is open on the official website of the AHRQ for free use,^1^ which can be used for the overall evaluation of teamwork skills and is suitable for teamwork under realistic situations. TPOP scoring items can be widely used in various medical teamwork situations. The TPOP observation scale includes: (1) Team organization (5 questions in total); (2) Leadership (6 questions in total); (3) Situation monitoring (4 questions in total); (4) Mutual support (5 questions in total); (5) Communication (5 questions in total): 1 (very poor) ~ 5 (excellent; Lin et al., 2018).

### Statistical analysis

This study is based on the fact that the two groups have never experienced high-fidelity simulation situation training in the past. All participants have nurse practitioner licenses and have little clinical experience. In order to strengthen the purpose and clinical significance of this study, statistical analysis was used to test the differences between pre-test and post-test of cognition, skills, attitude, self-efficacy, and teamwork in clinical assessment and management of dyspnea after the intervention of the situational simulated dyspnea teaching program. The data were analyzed by SPSS for descriptive statistics (number, percentage, and standard deviation) and inferential statistics (independent sample *t*-test, repeated-measures ANOVA). Then, generalized estimating equation (GEE) repeated measures scores were being made, and the statistical differences between pre- and post-test results were assessed by each measure scores. The GEE formula of the regression is as follows:

Variable score = Intercept ± (pre-test groups [pre-clinical nurses and NPGY]) (group) ± NPGY time (pre-test-post-test) ± Interaction between pre-test group and NPGY time (Group × Time).

## Results

### Subjects’ background information

A total of 135 members participated in the high-realistic situation simulation dyspnea teaching program, with an average age of 21.96 years old and a standard deviation of 1.44 years; 125 females, accounting for 92.59%; 10 males, accounting for 7.41%; educational level: bachelor’s degrees. The majority are 88 college junior and senior students, accounting for 65.18%; followed by 29 persons in university, accounting for 21.48%. Nine persons are from a 4-year college, accounting for 6.67%. Nine persons in junior college, accounting for 6.67%. There are 69 NPGY nurses, accounting for 51.11%; 66 pre-clinical nurses, 48.89%; 66 without clinical experience, 48.89%; 25 with internal medicine experience, 18.52%; and 44 with surgical experience, 32.59%. The average of clinical experience is 4.24 months.

### The learning effect of the high-realistic situation simulation of dyspnea teaching program

In pre-test, the pre-clinical Nurses’ cognitive and attitude scores were higher than that of NPGY nurses, reaching a significant difference (*t* = −3.624, *p* < 0.001 and − 6.926, *p* < 0.001) with independent sample *t*-test. However, NPGY nurses’ skill, self-efficacy, and teamwork pre-test scores were significantly higher than that of pre-clinical nurses (*t* = 2.357, *p* < 0.05; *t* = 2.442, *p* < 0.05; *t* = 3.062, *p* < 0.01). All participants have no experiences with high-realistic situation simulation dyspnea teaching program in the past. Yet, these results are not the main purpose of this study. Therefore, the results, showed that after the intervention of the high-realistic situation simulation dyspnea teaching program, reached significant difference, both in pre-test and post-test, in the learning effectiveness of nurses’ group and the pre-clinical nurses’ group.

In addition, each variable was determined by two-way repeated-measures ANOVA to compare the difference between the NPGY and the pre-clinical nurses group. For NPGY and nursing students after the intervention of the high-realistic situation simulation dyspnea teaching program, the difference between the pre- and post-tests of each variable is described as follows:

In cognition average scores of the pre- and post-test of NPGY nurses were 15.35 points and 18.30 points (*F* = 65.43, *p* < 0.001), respectively. Those of the pre-clinical nurses were 17.05 points and 18.97 points respectively(*F* = 23.15, *p* < 0.001), the post-test is better than the pre-test in both groups, and it reaches a statistically significant difference (Table 2).

**Table 2:** Analysis of the difference between the pre-test and the post-test of the high-realistic situation simulation of dyspnea teaching program (*N* = 135).

Item	NPGY (*n* = 69)				Pre-clinical nurses (*n* = 66)				Total (*n* = 135)			
	Pre-test	Post-test	*F*	*p*	Pre-test	Post-test	*F*	*p*	Pre-test	Post-test	*F*	*p*
	Mean ± SD				Mean ± SD				Mean ± SD			
Cognition	15.35 ± 2.80	18.30 ± 2.65	65.43	<0.001***	17.05 ± 2.63	18.97 ± 2.29	23.15	<0.001***	16.18 ± 2.84	18.63 ± 2.49	71.48	<0.001***
Attitude	23.17 ± 6.21	32.25 ± 4.14	91.33	<0.001***	28.83 ± 2.69	30.76 ± 4.20	13.88	<0.001***	25.94 ± 5.58	31.52 ± 4.22	74.06	<0.001***
Skills	22.61 ± 1.05	47.86 ± 3.87	16360.55	<0.001***	22.14 ± 1.28	42.89 ± 1.42	7552.73	<0.001***	22.38 ± 1.18	45.43 ± 3.84	9789.79	<0.001***
Self-efficacy	11.26 ± 3.45	14.96 ± 2.77	116.81	<0.001***	10.14 ± 1.63	14.24 ± 1.83	247.64	<0.001***	10.71 ± 2.76	14.70 ± 2.37	312.29	<0.001***
Teamwork	11.51 ± 0.56	20.30 ± 1.35	2768.46	<0.001***	11.15 ± 0.77	16.82 ± 0.72	1224.47	<0.001***	11.33 ± 0.69	18.60 ± 2.06	1842.66	<0.001***

*p-values relate to repeated-measures ANOVA. *p* < 0.05 was considered statistically significant (*p < 0.05, **p < 0.01, ***p < 0.001).

The average pre-test and post-test scores of NPGY nurses’ attitudes were 23.17 points and 32.25 points respectively, and the average scores of Pre-clinical Nurses’ attitudes pre-test and post-test were 28.83 points and 30.76 points, respectively. In both cases, the post-test was better than the pre-test, with a statistically significant difference (*F* = 91.33, *p* < 0.001 and *F* = 13.88, *p* < 0.01, respectively).

In formative clinical examination with OSCE, the average scores of the pre-test and post-test of NPGY nurses’ skills were 22.61 points and 47.42 points respectively(*F* = 16360.55, *p* < 0.001). The pre-clinical nurses’ skills’ pre-test average scores were 22.14 points, with a score of 42.89 points in post-test *(F*=,7552.73, *p* < 0.001). The post-test was significantly better than the pre-test between two groups. NPGY nurses’ self-efficacy pre-test and post-test average scores were 11.26 points and 14.95 points, respectively (*F* = 116.81, *p* < 0.001).

The average scores of pre-clinical nurses’ self-efficacy pre-test and post-test were 10.14 points and 14.24 points, (*F* = 247.64, *p* < 0.001) respectively. The post-test was better than the pre-test, and it reached a statistically significant difference in both groups.

In pre-test and post-test, the NPGY nurses’ teamwork average scores were 11.51 points and 20.30 points, respectively (*F* = 2768.4, *p* < 0.001). Of the pre-clinical nurses, the teamwork average scores were 11.15 points and 16.82 points (*F* = 1224.47, *p* < 0.001), respectively. All the post-tests were better than the pre-tests, and it reached a statistically significant difference.

### Learning effective difference between pre-test and post-test

Each variable was determined by two-way repeated-measures ANOVA to test the interaction between time and group. Further comparison of the difference between the pre- and post-test progress scores of NPGY nurses and pre-clinical nurses, the pre- and post-test progress scores of NPGY nurses are significantly higher than those of pre-clinical nurses (*p* < 0.001). In post-test, the cognition average scores was 18.63 ± 2.49, it better than the pre-test 16.18 ± 2.84 (*F* = 71.48, *p* < 0.001).

In pre- and post-test time points, all variable average scores of post-test were better than pre-test including the cognition average scores 18.63 ± 2.49 versus 16.18 ± 2.84 (*F* = 71.48, *p* < 0.001), attitudes average scores 31.52 ± 4.22 versus 25.94 ± 5.58 (*F* = 74.06, *p* < 0.001), skill average scores 45.43 ± 3.84 versus 22.38 ± 1.18 (*F* = 9789.79, *p* < 0.001), self-efficacy average scores 14.70 ± 2.37 versus 10.71 ± 2.76 (*F* = 312.29, *p* < 0.001), and teamwork average scores 18.60 ± 2.06 versus 11.33 ± 0.69 (*F* = 1842.66, *p* < 0.001).

### Cognition, skills, attitude, self-efficacy, and teamwork before and after GEE analysis results

The GEE are further used to test the differences between NPGY and pre-clinical nurses before and after intervention in the high-realistic situation simulation dyspnea teaching program, as follows (Table 3):

**Table 3:** GEE analysis of the difference in intervention effectiveness of the high-realistic situation simulation of dyspnea teaching program between the two groups.

Variables	*B*	*SE*	*X^2^*	*p*
Comparison of Cognitive Score^a^				
Intercept	15.348	0.335	2101.521	<0.001
Pre-test groups (pre-clinical nurses and NPGY)	1.689	0.464	13.368	<0.001
NPGY time (pre-test-post-test)	2.957	0.363	66.387	<0.001
Interaction between pre-test group and NPGY time	−1.032	0.573	3.241	0.072
Comparison of Skill Score^b^				
Intercept	22.609	0.125	32694.393	<0.001
Pre-test groups (pre-clinical nurses and NPGY)	−0.472	0.200	5.589	0.018
NPGY time (pre-test-post-test)	24.812	0.193	16601.144	<0.001
Interaction between pre-test group and NPGY time	−4.054	0.301	181.863	<0.001
Comparison of attitude score^c^				
Intercept	23.174	0.742	975.911	<0.001
Pre-test groups (pre-clinical nurses and NPGY)	5.659	0.811	48.68	<0.001
NPGY time (pre-test-post-test)	9.072	0.942	92.67	<0.001
Interaction between pre-test group and NPGY time	−7.148	1.125	40.35	<0.001
Comparison of self-efficacy score^d^				
Intercept	11.261	0.412	747.833	<0.01
Pre-test groups (pre-clinical nurses and NPGY)	−1.125	0.457	6.050	0.014
NPGY time (pre-test-post-test)	3.696	0.340	118.527	<0.01
Interaction between pre-test group and NPGY time	0.592	0.445	1.773	0.183
Comparison of teamwork score^e^				
Intercept	11.507	0.067	29672.636	<0.001
Pre-test groups (pre-clinical nurses and NPGY)	−0.356	0.115	9.516	0.002
NPGY time (pre-test-post-test)	8.797	0.166	2809.169	<0.001
Interaction between pre-test group and NPGY time	−3.130	0.201	241.622	<0.001

*p < 0.05 was considered statistically significant. General estimation equation of SAS was used to conduct repeated measuring analysis. ^a^Cognitive score = 15.348–1.689 (group) + 2.957 (time) − 1.032 (Group × Time). ^b^Skill core = 22.609 – 0.472 (group) + 24.812 (time) − 4.054 (Group × Time). ^c^Attitude score = 23.174 + 5.659 (group) + 9.072 (time) − 7.148 (Group × Time). ^d^Self-efficacy score = 11.261 – 1.125 (group) + 3.696 (time) + 0.592 (Group × Time). ^e^Teamwork score = 11.507 – 0.356 (group) + 8.797 (time) − 3.130 (Group × Time).

In the cognitive pre-test, the cognitive score of pre-clinical nurses was higher than that of NPGY nurses; however, further comparing the pre- and post-test difference between pre-clinical nurses and NPGY, the cognitive scores of pre-clinical nurses changed 1.032 points less than that of NPGY, *p* = 0.072, with no significant difference in cognitive improvement.

Before the intervention, the pre-clinical nurses had 5.659 points, significantly higher than the NPGY attitude score. After the intervention, NPGY nurses’ post-test attitude was 9.072 points higher than the pre-test score. Moreover, the difference between pre-clinical nurses and NPGY nurses before and after the test showed that the attitude score of nursing students was 7.148 points less than that of NPGY, and reached a statistically significant level (*p* < 0.001). The results showed that for the high-realistic situation simulation of breathing difficulties teaching program, NPGY’s attitude is better than that of nursing students.

Prior to pre-test, the clinical skill score of pre-clinical nurses was 0.472 points, statistically significantly lower than that of NPGY. Between NPGY clinical skills before and after testing, the difference of post-test and pre-test was increasing by 24.812, and reached a statistically significant difference. Comparing the pre- and post-test difference between pre-clinical nurses and NPGY, the skill change of pre-clinical nurses was 4.054 points less than that of NPGY (*p* < 0.001), showing that the high-realistic situation simulation of dyspnea teaching program intervention in NPGY skills is better than in that of pre-clinical nurses.

After the intervention, the post-test score of NPGY self-efficacy was 3.696 points higher than that of the previous test, and a statistical significance was reached. Comparing the difference between pre-clinical nurses and NPGY nurses before and after the intervention, the self-efficacy score of n pre-clinical nurses changed 0.592 points more than that of NPGY nurses. No statistically significant level (*p* = 0.183) is reached.

Before the intervention of the high-realistic situation simulation of dyspnea teaching program, the score of teamwork skill of pre-clinical nurses was lower than NPGY. After the intervention, NPGY’s post-test team score was higher than the pre-test 8.797 points, and reached a statistically significant level. Before and after the intervention, the change in the teamwork skill score of pre-clinical nurses was 3.130 points lower than that of NPGY, and reached a statistically significant level (*p* < 0.001). The results indicated that the intervention of the high-realistic situation simulation dyspnea teaching plan improved NPGY’s teamwork, better than that of pre-clinical nurses.

**Figure 3:**
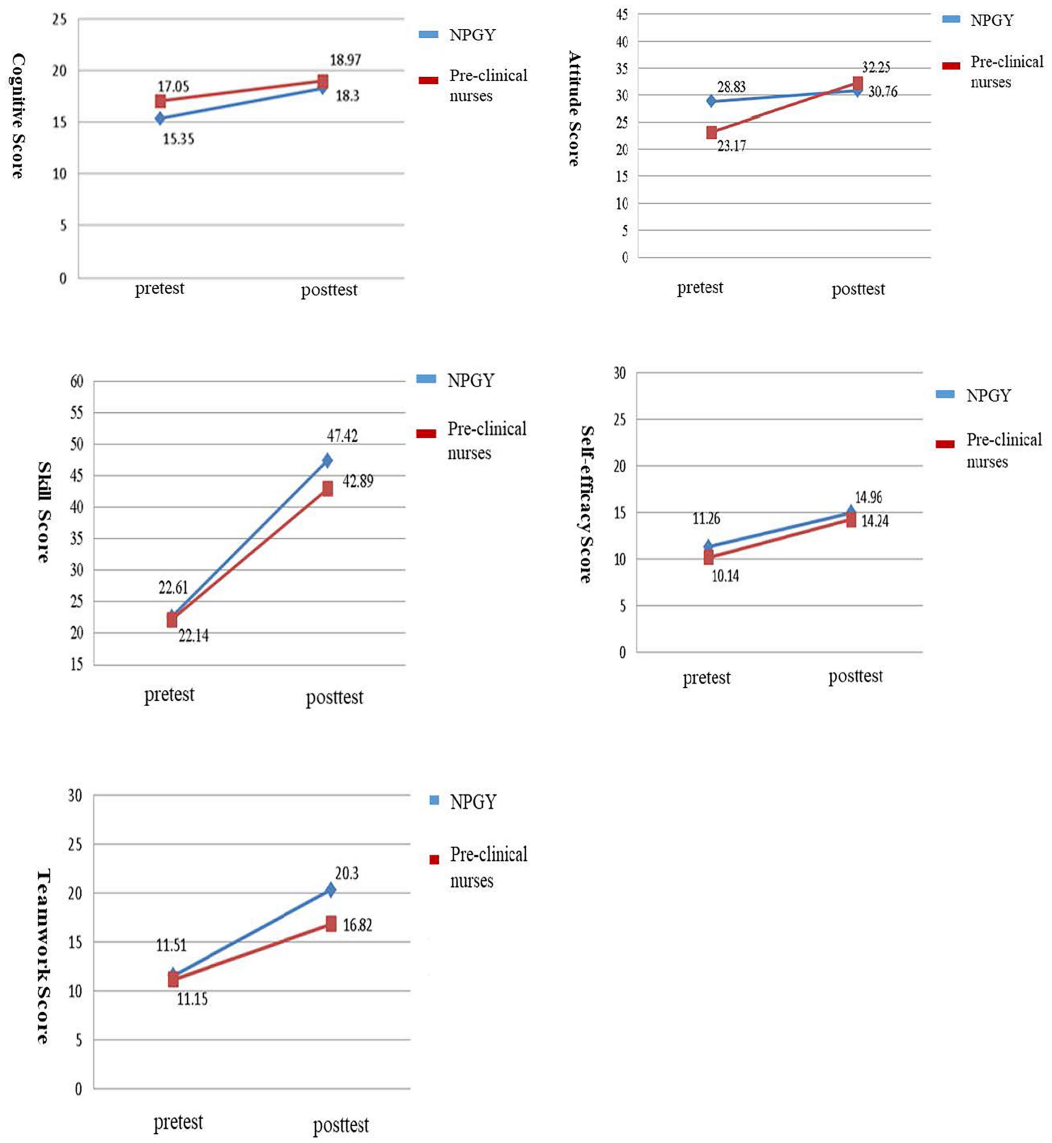
Change of cognitive, attitude, skill, self-efficacy and teamwork score in NPGY and pre-clinical nurses as assessed by GEE.

The average cognitive scores for the NPGY were 15.35 for pre-test and 18.3 for post-test. And average cognitive scores for the pre-clinical nurses were 17.05 for pre-test and 18.97 for post-test. The average attitude scores for the NPGY were 28.83 for pre-test and 30.76 for post-test. And average attitude scores for the pre-clinical nurses were 23.17 for pre-test and 32.25 for post-test. The average skill scores for the NPGY were 22.61 for pre-test and 47.42 for post-test. And average skill scores for the pre-clinical nurses were 22.14 for pre-test and 42.89 for post-test. The average self-efficacy scores for the NPGY were 11.26 for pre-test and 14.96 for post-test. And average self-efficacy scores for the pre-clinical nurses were 10.14 for pre-test and 14.24 for post-test. The average teamwork scores for the NPGY were 11.51 for pre-test and 20.3 for post-test. And average teamwork scores for the pre-clinical nurses were 11.15 for pre-test and 16.82 for post-test (Figure 3).

## Discussion

Based on the high-realistic patient simulation (HFPS) teaching method and the real clinical situation of the teaching plan, this research was well-organized and effectively conducted. The teacher uses the SimMan 3G and sets the heartbeat, lung sounds, breathing rate, skin color, movement, and voice functions of the simulator according to the teaching program. For the clinical intervention measures of the research objects, the instructor changes the parameters, provides students with realistic dyspnea simulation experience teaching, and allows the learners to practice dyspnea assessment and clinical treatment. Highly realistic patient simulation teaching intervention is similar to Kapucu (2017), McRae et al. (2017), and Smallheer et al. (2018). Our results show that the post-tests of “cognition,” “attitude” and “skills” of the NPGY group and the nursing student group are better than the pre-test, reaching statistically significant differences. The results, similar to the Kapucu (2017) study, used high-realistic SimMan and real emergency room scenes to conduct chest trauma emergency treatment training for third-year nursing students in the university. Interview data were collected, indicating the results beneficial to students’ skills and self-confidence. Smallheer et al. (2018) selected licensed nursing students with little clinical experience, and designed a total of 3 independent basic critical care simulation activities, including: abnormal heart rhythm, respiratory system complications, and bedside care of critical patients, the introduction of realistic learning strategies. Students describe that the simulation allows them sufficient time to think critically in a safe environment, enables them to have greater confidence, and think critically before becoming intensive care nurses. Vandyk et al. (2018) searched for the use of psychiatric simulation education from 2004 to 2015, and included 32 articles and a total of 2,724 people were included in the study. The study concluded that through the simulation education program of realistic situations, students’ knowledge, empathy, communication, and self-confidence were improved.

The Waxman et al. (2019) simulation can replace 50% of clinical time, improve skills, communication, and teamwork. Tamaki et al. (2019) conducted a randomized controlled trial on the effectiveness of hospice care simulation in nursing education, with 38 nursing students participating. A self-developed questionnaire was used to measure the OSCE. It was used to evaluate the effectiveness. Assessing the “clinical skills” results, the simulation group’s knowledge, physical assessment and psychological nursing skills, and self-confidence scores all improved significantly (*p* < 0.001). In addition, self-efficacy refers to the confidence and expected behavior that one feels capable of when interacting with the environment and can successfully accomplish tasks. Our results showed that the “self-efficacy” of the NPGY group and the nursing students group are better than in the pre-test, with a statistically significant difference. Kapucu (2017) uses high-realistic SimMan and real emergency scenarios. Seven third-year nursing students were trained in the emergency treatment of chest trauma. The results showed that simulated learning can enhance the confidence of the students. Shu et al. (2019) use realistic teaching to improve the assessment and treatment of abnormal endotracheal events. In addition to effectively improving knowledge and skills, the students’ self-assessment and teachers’ evaluation of students’ self-confidence are both “very confident.”

In terms of teamwork, the post-test of the NPGY group and the nursing students group is better than the pre-test, with a statistically significant difference. The research results show that the high-realistic situational simulation of the dyspnea teaching program can effectively improve the effectiveness of participants’ “teamwork.” Vandyk et al. (2018) searched and reviewed 32 articles from 2004 to 2015. With 2,724 participants involved, they found that through the simulation education program of realistic situations, participants’ communication and cooperation were statistically significantly improved. The students could better understand the role function and cooperation relationship of each medical team before becoming NPGY nurses. Liu (2016) uses empirical concepts to explore the effectiveness of realistic nursing situational teaching in situational awareness, clinical ability, teamwork and communication, and critical thinking. A total of 15 English full-text articles are included. The integrated literature results show that simulated situational teaching and simulated situations can effectively improve the clinical ability of clinicians and students, their teamwork and communication, and interprofessional communication.

Through the independent sample t-test, the difference between the two groups’ (difference of) “cognition” and “self-efficacy” did not reach a significant difference. For “attitude,” “skills” and “teamwork,” the NPGY pre- and post-test progress score is higher than that of nursing students, with a significant difference. Further, through GEE analysis and verification, after the intervention of the high-realistic situation simulation dyspnea teaching program, the difference between nursing students and the NPGY pre- and post-tests was compared. The pre- and post-test difference in the “cognition” and “self-efficacy” between nursing students and NPGY was still not at a significant level. NPGY group’s “attitude,” “skills” and “teamwork” scores are higher than the nursing students, and reach a statistically significant level. According to the above statistical results, after the intervention of the high-realistic situation simulation dyspnea teaching program, the learning effect of “skills,” “attitude” and “teamwork” in the NPGY group is better than that of the nursing student group. However, no significant difference between “cognition” and “self-efficacy” in learning effectiveness was found. Although some studies use realistic teaching methods to improve the learning effectiveness of NPGY or newcomers, or separately discuss the effectiveness of applying them to nursing students, related literature on learning effectiveness between NPGY and nursing students remains none. We proposed that the NPGY group has entered clinical work to practice, and may have experience dealing with the actual dyspnea situation of clinical patients. Through the high-realistic situation simulation dyspnea teaching program, its skills, attitudes and teamwork have improved. NPGY are better than nursing students who have little experience in treatment of dyspnea.

It is inferred from the research results, which can provide a reference for the curriculum design of nursing schools. The timing of intervention in high-realistic situational teaching courses should be arranged and established after students have the basis of medical and surgical internships and emergency and severe illness-related internships. High-realistic situational courses can focus on clinical practice. The most frequently encountered difficult handling situations can also improve the cognition, attitude, clinical skills, and self-confidence of nursing students when they are in line with clinical practice before graduation.

## Conclusion

Compared with the traditional teaching mode, simulation teaching hardware and equipment are expensive to build and maintain, and the teaching preparation process is time-consuming and labor-intensive. Teachers need to have the ability to use equipment and situational design, and continue to be proficient in new teaching skills, with higher teaching loads. Therefore, it has not been widely used in nursing educational systems. Our results show that high-realistic simulation teaching strategies can improve the learning goals of pre-clinical nurses and NPGY students’ cognition, attitudes, and skills, and help students develop their professional qualities and abilities required for clinical practice. Simulation is indeed an indispensable strategy for nursing education. The high-realistic situation simulation teaching program can provide learners with a safe learning environment to practice and improve various skills. Through the practice simulation situation, important clinical experience is replicated, and the situations that may occur in practice are repeated to deepen the development of learning and ability. Applied to the design of NPGY on-the-job education, it is recommended that the courses can be added to the training courses for new recruits entering the workplace for 1 to 3 months. The same focus is on the most common clinically difficult situations, which can improve their clinical ability, attitude, and teamwork. Furthermore, the application of high-fidelity simulation teaching on pre-graduation nursing students can help to resolve the lack of clinical reasoning education, the gap between post-graduation learning and practice. Therefore, the application training helps to promote students’ responsibilities, and abilities, and shorten their adaptation period in the workplace so as to solve confronted problems such as clinical turnover rate. In the future, high-realistic situational teaching can be designed in NPGY and pre-clinical nursing students training for different levels of clinically difficult situations to improve the learning effectiveness of clinical nursing staff. The research results, therefore, can provide future school teaching and hospitals to arrange NPGY on-the-job education as a reference. Ultimately, the limitation of this study is that it failed to recruit staffs from other medical centers and nursing universities. If qualitative interviews can be used to supplement information that cannot be presented by quantitative data, it is possible to better understand the feelings and needs of learners.

## Data availability statement

The original contributions presented in the study are included in the article/supplementary material, further inquiries can be directed to the corresponding author.

## Ethics statement

The studies involving human participants were reviewed and approved by CMUH108-REC3-06, Research Ethics Committee, China Medical University & Hospital, Taichung, Taiwan. The patients/participants provided their written informed consent to participate in this study.

## Author contributions

Y-MS, Y-HL, and P-HL: conceptualization and methodology. Y-HL and P-HL: investigation and data creation. Y-HL and Y-MS: writing. Y-MS: draft preparation, review and editing, and supervision. All authors contributed to the article and approved the submitted version.

## Conflict of interest

The authors declare that the research was conducted in the absence of any commercial or financial relationships that could be construed as a potential conflict of interest.

## Publisher’s note

All claims expressed in this article are solely those of the authors and do not necessarily represent those of their affiliated organizations, or those of the publisher, the editors and the reviewers. Any product that may be evaluated in this article, or claim that may be made by its manufacturer, is not guaranteed or endorsed by the publisher.

## Footnotes

^1^ https://www.ahrq
